# A Catalytic Role of XoxF1 as La^3+^-Dependent Methanol Dehydrogenase in *Methylobacterium extorquens* Strain AM1

**DOI:** 10.1371/journal.pone.0050480

**Published:** 2012-11-27

**Authors:** Tomoyuki Nakagawa, Ryoji Mitsui, Akio Tani, Kentaro Sasa, Shinya Tashiro, Tomonori Iwama, Takashi Hayakawa, Keiichi Kawai

**Affiliations:** 1 Faculty of Applied Biological Science, Gifu University, Gifu, Japan; 2 Department of Biochemistry, Faculty of Science, Okayama University of Science, Kita-ku, Okayama, Japan; 3 Institute of Plant Science and Resources, Okayama University, Kurashiki, Okayama, Japan; 4 Tokai Gakuin University, Kakamigahara, Gifu, Japan; Louisiana State University and A & M College, United States of America

## Abstract

In the methylotrophic bacterium *Methylobacterium extorquens* strain AM1, MxaF, a Ca^2+^-dependent methanol dehydrogenase (MDH), is the main enzyme catalyzing methanol oxidation during growth on methanol. The genome of strain AM1 contains another MDH gene homologue, *xoxF1*, whose function in methanol metabolism has remained unclear. In this work, we show that XoxF1 also functions as an MDH and is La^3+^-dependent. Despite the absence of Ca^2+^ in the medium strain AM1 was able to grow on methanol in the presence of La^3+^. Addition of La^3+^ increased MDH activity but the addition had no effect on *mxaF* or *xoxF1* expression level. We purified MDH from strain AM1 grown on methanol in the presence of La^3+^, and its N-terminal amino acid sequence corresponded to that of XoxF1. The enzyme contained La^3+^ as a cofactor. The Δ*mxaF* mutant strain could not grow on methanol in the presence of Ca^2+^, but was able to grow after supplementation with La^3+^. Taken together, these results show that XoxF1 participates in methanol metabolism as a La^3+^-dependent MDH in strain AM1.

## Introduction

Methylotrophs are microorganisms with the ability to utilize reduced C_1_-compounds, such as methane, methanol and methylamine as their sole carbon and energy source. They are ubiquitous in nature, and some of them are well-known plant epiphytes [1], [2]. Among them, the genus *Methylobacterium*, an aerobic facultative methylotrophic α-proteobacterium, is one of the most abundant bacterial genera in the phyllosphere [3]–[5], with a titer between 10^4^ and 10^7^ colony-forming units (CFU) per gram fresh weight of plant material [6]. Over the past few decades, considerable work has been done on the methylotrophy of *Methylobacterium* and their symbiosis with plants, as *Methylobacterium* can metabolize the methanol released by plants and may also grow on other plant-derived carbon compounds [7]–[9]. *M. extorquens* strain AM1 serves as an important model organism for studying methylotrophy in bacteria [10], [11], and the genome sequence of the strain is available [12].

In the methylotrophic metabolism of *Methylobacterium*, methanol is first oxidized to formaldehyde via methanol dehydrogenase (MDH) in the periplasm [13], [14]. MDH is a heterotetrameric protein (α2β2) consisting of two 66-kDa large subunits (MxaF) and two small 8.5-kDa subunits (MxaI) [15], and contains Ca^2+^ and pyrroloquinoline quinone (PQQ) as a prosthetic group in the active site [15], [16]. MxaF and MxaI are encoded by *mxaFI* genes located in the large *mxa* gene cluster [17], and both are essential for growth on methanol, as the loss of these genes in strain AM1 eliminates virtually all methanol dehydrogenase activity [18], [19].

The genome of strain AM1 contains several homologs of MxaF, one of which is named XoxF1 [20]. XoxF1 is predicted to be a PQQ-dependent periplasmic MDH exhibiting 50% sequence identity to MxaF. Recently, Schmidt *et al*. reported that XoxF1 was found to be strongly expressed in bacterial phyllosphere communities [1], and that the *xoxF1*-deleted strain was less competitive than the wild-type during colonization in the phyllosphere, although XoxF1 had low MDH activity in strain AM1 [21]. Skovran *et al*. showed that the double mutant of both *xoxF* homologs (*xoxF1* and *xoxF2*) was unable to grow on methanol and that the expression of the two-component regulatory systems MxcQE and MxbDM required for activation of the *mxa* genes is repressed in the double mutant strain [22]. From these facts, it is clear that XoxF functions in the regulation of methanol metabolism, but its catalytic function as an MDH has not been clear.

In our previous work, we showed that lanthanum (La), cerium (Ce), and praseodymium (Pr), all of which are belong to the rare earth elements (REE), increased MDH activity in cell extracts of *M. radiotolerans* and the non-methylotrophic bacteria *Bradyrhizobium* sp. [23], [24]. Moreover, the MDHs purified from the cells grown in media containing these metal ions corresponded to XoxF1, while the MDH purified from Ca^2+^-grown cells corresponded to MxaFI [23], [24]. These results indicate that the MDHs dependent on La^3+^, Ce^3+^ or Pr^3+^ are products of *xoxF* and that these ions may have important physiological roles in C_1_ metabolism.

The REEs are a group of 17 elements, specifically, 15 lantanoids plus Sc and Y, and are widely dispersed among many primary and secondary minerals, such as phosphates, carbonates, fluorides, and silicates, especially pegmatites, granites, and related metamorphic and igneous rocks [25]. They are regarded as “the vitamins of modern industry”, since many of them are utilized in a wide range of industrial products such as glass, catalysts, alloys, ceramics, and magnets. As for their effects on life forms, the REEs have not been characterized as either essential or strongly toxic elements in the environment [26], although some have negative effects as inhibitors of several enzymes and proteins [27]–[30], and some exert positive effects as growth promoters for various crops [27].

In this study, using *M. extorquens* strain AM1 as a model organism to investigate REEs-dependent methylotrophy, we set out (i) to see whether La^3+^ is involved in methylotrophic growth of the strain, (ii) to assess whether the strain has REE-dependent MDH activity, (iii) to identify the gene encoding REE-dependent MDH, and (iv) to validate the role of XoxF1 and La^3+^ in methanol metabolism. Our results suggest that XoxF1 is a La^3+^-dependent functional MDH that may participate in methanol metabolism.

## Results

### *M. extorquens* strain AM1 has a methanol-oxidation system independent of Ca^2+^

**Figure 1:**
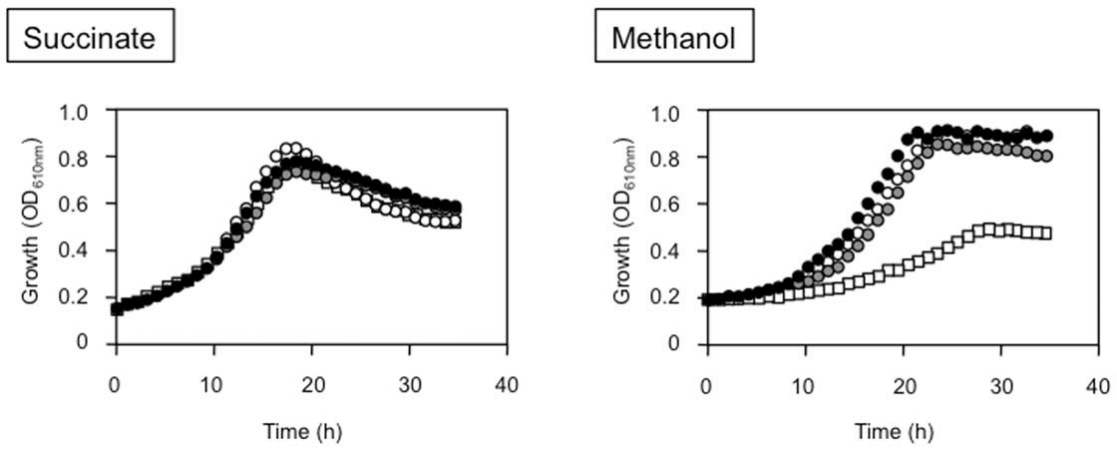
Growth of the wild-type strain AM1 on methanol or succinate media supplemented with Ca^2+^ or/and La^3+^. The growth of strain AM1 on methanol or succinate media supplemented with Ca^2+^ (black circle), with La^3+^ (gray circle), with Ca^2+^+La^3+^ (white circle) and without Ca^2+^ and La^3+^ (white square). The concentration of Ca^2+^ and La^3+^ in the medium are 30 µM each. Points on the graphs depict average data from three biological replicates.

Although MDH activity in *Methylobacterium* species has been shown to depend on Ca^2+^ [14], the growth of these strains on methanol without Ca^2+^ has never been examined. In our previous work, we showed that some REEs increased MDH activity in *M. radiotolerans* and the non-methylotrophic *Bradyrhizobium* sp. [23], [24]. These facts suggest that REEs may have some roles as activators or inducers of MDH. Thus, we examined whether *M. extorquens* strain AM1 could grow on methanol in the presence of La^3+^ instead of Ca^2+^. As shown in Fig. 1, strain AM1 could grow normally in methanol/Ca^2+^ medium. In methanol medium without Ca^2+^ and La^3+^, the strain showed very slow growth, because the medium contained a small amount of Ca^2+^ (0.867 µM). In methanol media containing La^3+^ and not Ca^2+^, the strain grew as well as it did in methanol/Ca^2+^ medium, and the addition of La^3+^ to methanol/Ca^2+^ medium had no effect on the growth of strain AM1 (Fig. 1). On the other hand, strain AM1 did not show any growth defect in succinate media even without Ca^2+^ and La^3+^. Ca^3+^ and La^3+^ have an important role in methanol metabolism but not in succinate metabolism, and strain AM1 has a novel methanol-metabolic pathway that depends on La^3+^ and not Ca^2+^.

### XoxF1 is a functional La^3+^-dependent MDH

**Figure 2:**
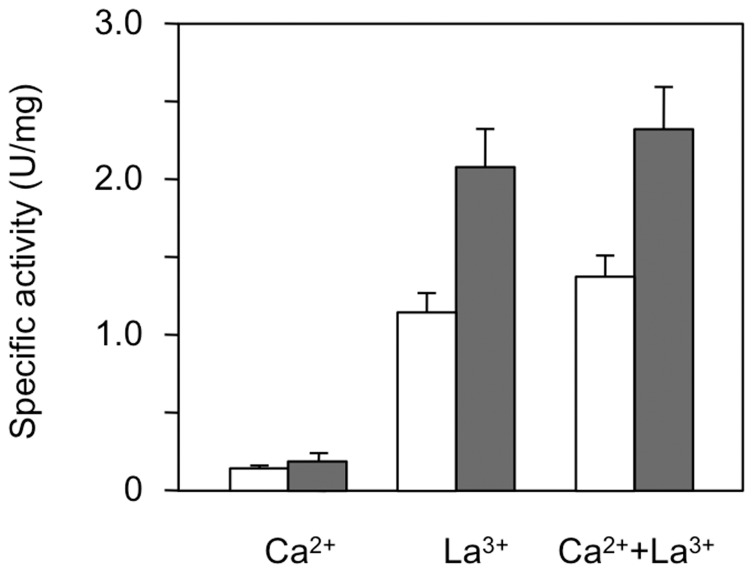
MDH activity in strain AM1 grown on the methanol and succinate media with Ca^2+^ and/or La^3+^. Cells were grown aerobically in succinate (white bar) and methanol (gray bar) media with 30 µM La^3+^ and/or Ca^2+^ at 30°C. Results are shown as means with standard deviations (*n* = 3).

The growth defect of strain AM1 in the methanol medium without Ca^2+^ was restored by the addition of La^3+^ to the medium. Next, in order to see whether MDH activity was induced by La^3+^, we measured MDH activity of strain AM1 grown on media containing La^3+^. When strain AM1 was grown in methanol media, MDH activity in the cell-free extract was ten times greater in methanol/La^3+^+Ca^2+^ medium than in methanol/Ca^2+^ medium, and cells grown in methanol/La^3+^ medium showed levels of MDH activity similar to those in cells grown in methanol/La^3+^+Ca^2+^ medium (Fig. 2). Cells grown on the succinate media also had enough MDH activity more than half of the activity in the methanol-grown cells, and the MDH activity induced on the succinate/La^3+^ medium was higher than that induced on the succinate/Ca^2+^ medium, as well as the methanol grown cells. There are two possible explanations for this positive effect of La^3+^: one is that La^3+^ enhances MDH gene(s) expression and the other is that La^3+^ activates MDH protein.

**Figure 3:**
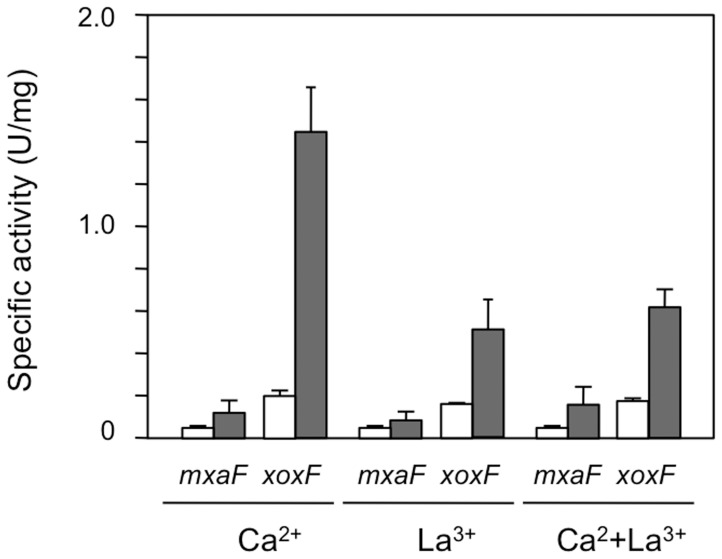
Expression of *mxaF-xylE* and *xoxF1-xylE* promoter fusions in *M. extorquens* strain AM1. The *mxaF* and *xoxF1* promoter activities were determined by measuring catechol 2,3-dioxygenase activity (XylE) in crude cell extracts of strains grown in methanol (black bar) or succinate (white bar) media containing 30 µM La^3+^ and/or Ca^2+^. Results are shown as means with standard deviations (*n* = 3).

To determine whether La^3+^ enhances MDH gene(s) expression, we quantified the gene expression levels of *mxaF* and *xoxF1* using cells harboring the *xylE* reporter gene regulated by the predicted promoter regions, which are 220- and 227-bp upstream sequences of the *MxaF* and *XoxF1* genes, respectively. The reporter activities regulated by the *mxaF* and *xoxF1* promoters were detected in all cells grown on methanol or succinate, and the *xoxF1* promoter of the cells grown on methanol/Ca^2+^ medium exhibited the highest expression activity (Fig. 3). The activities of both promoters on the methanol grown cells exhibited always higher than those on the succinate grown cells (Fig. 3). Moreover, expression activity of the *xoxF1* promoter was always greater than that of the *mxaF* promoter on any media, irrespective of the presence of La^3+^ and/or Ca^2+^. XylE activity was not detected in cells harboring the promoterless control plasmid pCM130, irrespective of the carbon sources, as reported previously [31]. These results show that the increase in MDH activity caused by La^3+^ is due not to an increased expression of MDH genes but rather to post-translational activation of MDH.

**Figure 4:**
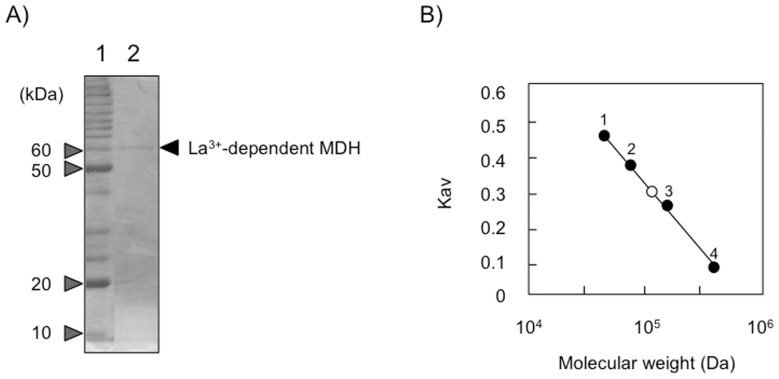
SDS–PAGE analysis (A) and molecular weight (B) of purified MDH from strain AM1 grown on methanol/Ca^2+^+La^3+^ medium. A, Lane 1, marker proteins; lane 2, purified MDH. B, Marker proteins were: 1, ovoalbumin (43 kDa); 2, conalbumin (75 kDa); 3, aldolase (158 kDa); and 4, ferritin (440 kDa).

We then purified MDH from strain AM1 cells grown in methanol/La^3+^+Ca^2+^ medium in order to identify the La^3+^-dependent MDH and to observe whether MxaF and XoxF are concurrently activated by La^3+^ and Ca^2+^ (Table 1). In all the purification steps, we observed only one fraction peak showing MDH activity (data not shown). The purified MDH had a specific activity of 10.0 U/mg of protein. The protein migrated as a single protein band on the SDS-PAGE gel with an apparent molecular mass of 61 kDa. A small protein corresponding to subunit β was not observed (Fig. 4), although the MDH purified from cells grown in methanol/Ca^2+^ medium showed two bands for α and β subunits (data not shown). Using gel chromatography with a Superdex G-200 GL column, the native molecular weight of the purified protein was estimated to be ca. 117 kDa (Fig. 4B). These results indicated that the purified MDH is a homodimer of only the α subunit. The purified enzyme contained 0.91 atoms of La^3+^ and 0.39 atoms of Ca^2+^ per dimer. After treatment with 50 mM EDTA, the La^3+^ and Ca^2+^ contents in the enzyme were shown to be 1.24 and 0.10 per dimer, respectively, suggesting that the La^3+^ is tightly bound to the enzyme. The N-terminal amino acid sequence of the MDH protein was NESVLKGVANPAEQVLQTVD, which was completely identical to 22–41 amino acid residues of the deduced amino acid sequence of the *xoxF1* ORF. The predicted cleavage site of the amino acid sequence of XoxF1 as predicted by SignalP version 4.0 [32] was Ala21-Asn22. This cleavage site coincides completely with the N-terminal amino acid sequence of the purified MDH. Taken together, our data show that the *xoxF1* gene encodes a functional La^3+^-dependent MDH and that XoxF1 may have a signal peptide for periplasmic localization.

**Table 1:** Purification scheme of the La^3+^-dependent MDH isolated from *M. extorquens* strain AM1.

Step	Total activity (Unit)	Specific activity (U/mg)	Purification (fold)	Yield (%)
Cell free extract	46	0.62	1.0	100
PD-10	33	0.74	1.2	71
Hi-trap SP HP Sepharose HP	15	14	22	32
MonoS 5/50 GL	4.5	10	17	18

### *XoxF1* encodes an essential MDH for the methylotrophy depending on La^3+^

**Figure 5:**
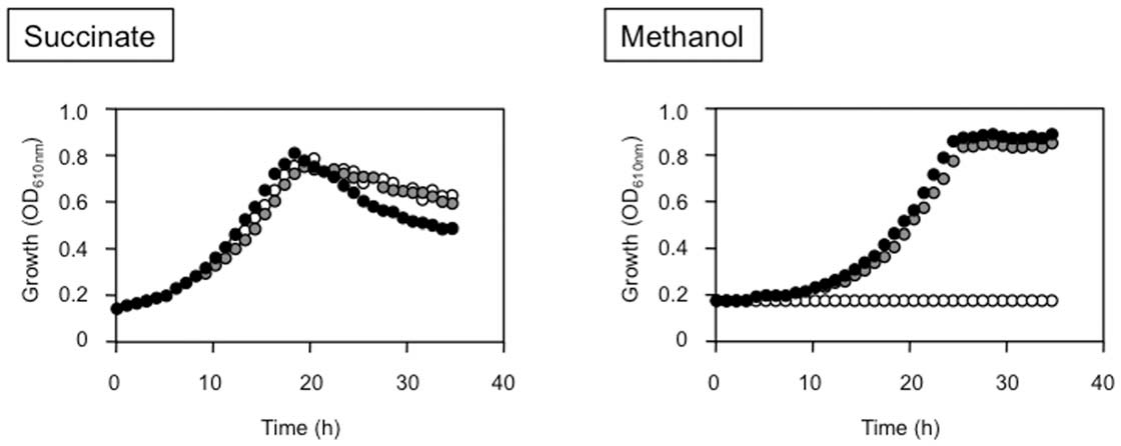
Phenotypic growth defects in strain Δ*mxaF* on methanol and succinate media. Growth on media containing Ca^3+^ (white circle), La^3+^ (gray circle), and Ca^2+^+La^3+^ (black circle). Graphs depict average data from three biological replicates.

We next examined the growth behavior and MDH induction patterns of an MxaF-disrupted mutant. In succinate media containing La^3+^ and/or Ca^2+^, strain Δ*mxaF* exhibited normal growth comparable to that of the wild-type strain (Fig. 5). Strain Δ*mxaF* could not grow on methanol at all in the presence of Ca^2+^ as previously reported [22], but its growth was restored by supplementation with La^3+^ even without Ca^2+^ (Fig. 5).

**Figure 6:**
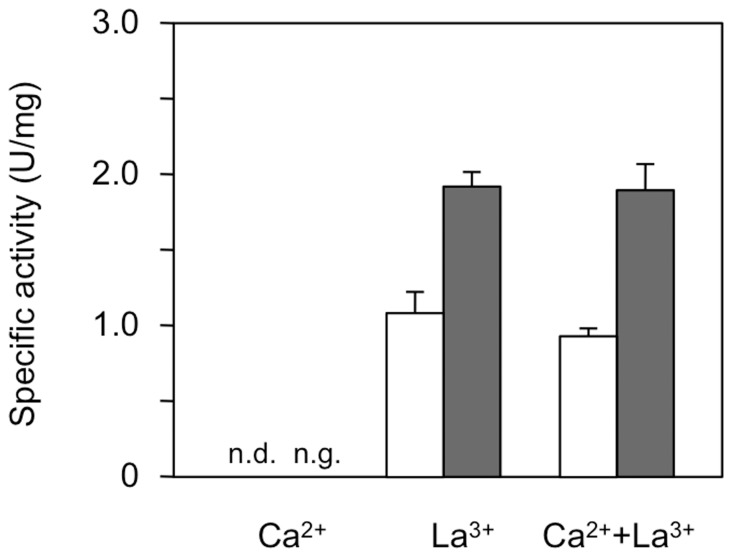
MDH activity in strain Δ*mxaF* grown on the methanol and succinate media with Ca^2+^ and/or La^3+^. Cells were grown aerobically in succinate (white bar) and methanol (gray bar) media with 30 µM Ca^3+^, La^3+^, and Ca^2+^+La^3+^ at 30°C. n.d.: not detected, and n.g.: no growth. Results are shown as means with standard deviations (*n* = 3).

Strain Δ*mxaF* grown in succinate/Ca^2+^ medium did not show detectable MDH activity (Fig. 6). Strain Δ*mxaF* grown in succinate or methanol media containing La^3+^, however, showed MDH activity comparable to that of the wild-type strain grown in succinate or methanol media containing La^3+^ (Fig. 6). These results suggest that the XoxF1 is able to function as the MDH in the cells in the presence of La^3+^, in place of MxaF, which explains the growth of the mutant on methanol in the presence of La^3+^.

## Discussion

*Methylobacterium* species and various other bacteria harbor *xoxF*, which is homologous to *mxaF*, encoding the large subunit of MDH. Over the last few years, the function of XoxF has been the subject of controversy. Using strain AM1, Schmidt *et al*. showed that XoxF1 had low activities of methanol and formaldehyde dehydrogenase activity [21], and the work by Skovran *et al*. suggested that XoxF1 and XoxF2 might have some roles in the expression of the MDH genes [22]. Nevertheless, the function and physiological role of XoxF in methanol metabolism is not yet completely understood.

In this work, we showed that XoxF1 from strain AM1 functions as a La^3+^-dependent MDH and has a role in La^3+^-dependent methanol metabolism of the strain, because (i) purified XoxF1 from the strain grown on methanol in the presence of La^3+^ contained La^3+^ with significant MDH activity, (ii) strain AM1 could grow on the methanol/La^3+^ medium even without Ca^2+^, and (iii) the growth defect of strain Δ*mxaF* was completely restored by supplementation with La^3+^. In our previous work, we have shown that MDHs purified from *M. radiotolerans* strain NBRC15690 grown in the presence of REEs and non-methylotrophic bacterium *Bradyrhizobium* sp. strain MAFF211645 have significant MDH activity and that the N-terminal amino acid sequences of both enzymes were identical to those of XoxF homologues [23], [24]. These facts suggest that XoxF1 in strain AM1 has an important physiological role as an MDH in the methanol metabolism in the presence of La^3+^, and that an REE-dependent methanol-metabolic pathway may be distributed among known methylotrophic bacteria as well as in other non-methylotrophic bacteria containing XoxF homologues.

It has been reported that XoxF1 purified from strain Δ*mxaF* harboring the pCM80-xoxF-His vector grown on Ca^2+^-containing medium exhibits low MDH activity (*V*_max_ value for methanol is 0.015 U/mg) [21]. La^3+^-dependent XoxF1, however, exhibited significant specific activity for methanol (10.0 U/mg), with levels over fifteen times higher than those in purified Ca^2+^-induced MxaFI from cells grown on Ca^2+^-containing medium (0.66 U/mg) (data not shown). Strain Δ*mxaF* grown in the methanol/Ca^2+^ medium had little MDH activity despite high expression levels of the *xoxF1* gene (Fig. 3). Moreover, Ca^2+^-induced XoxF1 was a monomer [21], while La^3+^-containing XoxF1 was a homo-dimer of α-subunits only (Fig. 4). Thus it can be hypothesized that La^3+^ can facilitate the dimerization of XoxF1 protein, which in the absence of La^3+^ is an inactive monomeric apo-enzyme. Similarly, since there was no fraction showing any MDH activity except for that containing XoxF1, MxaFI may be inactive in cells grown in the presence of La^3+^, although their hetero-tetramerization has not been examined (data not shown). It has been reported that Ca^2+^-dependent enzymes and other proteins, *e.g*., horseradish peroxidase, might be inhibited by La^3+^ [27]–[30]. Thus we hypothesize that La^3+^ may inhibit MxaFI posttranslational activation and/or its activity. Taken together, our work suggests that the posttranslational activation of XoxF1 and that of MxaFI require La^3+^ and Ca^2+^, respectively, and that strain AM1 has the ability to generate MDH, either XoxF1 or MxaF, depending on which metal is present, for methanol metabolism.

La^3+^ is one of the REEs, which are relatively abundant in the earth's crust (35 µg/g for La^3+^, 66 µg/g for Ce^3+^, and 40 µg/g for Nd^3+^); in fact, the abundance of Ce^3+^ is almost equal to those of much more commonly studied elements in the environment, such as Cu and Zn [26]. La^3+^ exists in all plants examined, with levels of around 0.178–3.1 µg/g dry mass in leaves, which is in the same range as Mn (0.5–15.6 µg/g dry mass) and Fe (1.33–2.5 µg/g dry mass) [25]. Meanwhile, Delmotte *et al*. have reported that XoxF is highly expressed in bacterial phyllosphere communities *in situ*, with a prevalence similar to that of MxaF as demonstrated by shotgun proteomics [1]. Therefore, the *Methylobacterium* species, as major plant epiphytes, would be readily able to access La^3+^ and Ca^2+^ on plant surfaces in the natural environment, and it is highly possible that XoxF1 is active on plant leaf surfaces together with MxaFI, because XoxF1 and MxaF are induced by methanol regardless of the presence of La^3+^ and/or Ca^2+^.

In this paper, we showed that XoxF1 is a functional MDH that depends on La^3+^. As far as we know, this is the first report of a metabolic pathway and enzyme dependent on an REE as a cofactor. Recently, XoxF was reported to be involved in a complex regulatory cascade of a MxcQE two-component system [22]. Taking our data together with these reports, it appears that XoxF may play a dual role in both regulation of MDH genes and catalysis of methanol oxidation.

## Materials and Methods

### Bacterial strains, media, and cultivation

*M. extorquens* strains and plasmids used in this study are described in Table 2. *M. extorquens* strains were cultivated in minimal salts (MS) media [33] supplemented with 0.5% methanol or 0.4% succinate as a carbon source. MS medium with 0.5% methanol is referred to as methanol/Ca^2+^ medium, methanol/Ca^2+^ medium containing 30 µM LaCl_3_ is referred to as methanol/Ca^2+^+La^3+^ medium, and MS medium with 0.5% methanol containing 30 µM LaCl_3_ instead of CaCl_2_ is referred to as Methanol/La^3+^ medium. For the cultivation of strain AM1 to purify La^3+^-dependent MDH, a 1/10 nutrient medium supplemented with 0.5% methanol and 30 µM LaCl_3_ was used [23]. In this medium, the content of Ca^2+^ was 31.8 µM. When appropriate, antibiotics were added at the following concentrations: tetracycline (Tc), 10 µg/ml, and kanamycin (Km), 50 µg/ml.

**Table 2:** Strains and plasmids used in this study.

Strain or plasmid		Description	Source or reference
Strains	AM1	Wild type (JCM 2805)	JCM
		Rif^r^ derivative (wild type)	18,19
	Δ*mxaF*	*mxaF*::Km	This study
Plasmids	pCM184	Allelic exchange suicide vector (Km^r^ Tc^r^ Ap^r^)	35
	pΔmxaF	pCM184 with *mxaF* upstream and downstream flanks (Km^r^ Tc^r^ Ap^r^)	This study
	pCM130	Promoter-less *xylE* fusion vector (Tc^r^)	31
	pRM246	pCM130 with *mxaF* promoter region (Tc^r^)	This study
	pRM248	pCM130 with *xoxF1* promoter region (Tc^r^)	This study

Cultivation of *M. extorquens* strains was done in 200 µl of MS media in 96 well round bottom microplates (Asahi Glass Co., Ltd., Chiba, Japan) at 28°C with reciprocal shaking, and growth was monitored by measuring the optical density at 610 nm in the a HiTS BioMicroplate reader (Scinics co, Ltd., Tokyo, Japan).

*Escherichia coli* strains DH5α and S17-1 [34] were routinely cultivated at 37°C in Luria-Bertani medium. The following antibiotic concentrations were used: tetracycline, 10 µg/ml, kanamycin, 50 µg/ml, and ampicillin, 100 µg/ml.

### Construction of null mutants

Null mutants were generated in *mxaF* using the allelic exchange vector pCM184 [35]. The following primers were used for the amplification of the up- and downstream regions of *mxaF*: upstream of *mxaF,* mxaFup-fw (5′-GAATTCTCACGATGCGGCACTTCGGATG-3′) and mxaFup-rv (5′-GAATTCTTCTCGACCTTCCACACCGTCTC-3′); downstream of *mxaF,* mxaFdn-fw (5′-GTTAACGGATGGACCACCTCGCCAAGGA-3′) and mxaFdn-rv (5′-GAGCTCTTCGCATCTGCCGTC, AGGCAGT-3′). Each PCR fragment was introduced into pCM184. The resulting allelic exchange vectors were introduced into *M. extorquens* strain AM1 via conjugation using *E. coli* strain S17-1. Mutations were confirmed by diagnostic PCR.

### Construction of promoter fusions

*mxaF* and *xoxF1* promoter fusions with *xylE* encoding catechol 2,3-dioxygenase were constructed in vector pCM130 [31]. The following primers were used for amplification of the promoter regions of *mxaF* and *xoxF1*: mxaF promoter, PmxaF-fw (5′-GGATCCGGTCAAGACGATGCCAATAC-3′) and PmxaF-rv (5′-AAGCTTCTCGGAAGTCATCCGAAGTG-3′); xoxF1 promoter, PxoxF1-fw (5′-GGATCCTTCGTTCAAGCTTCGGTTTC-3′) and PxoxF1-rv (5′-GCATGCTCATGGATTCCTCCGACAAG-3′). The resulting *P_mxaF_-xylE* and *P_xoxF1_-xylE* fusions were transferred into strain AM1 via conjugation.

### Preparation of crude extracts and enzyme assays

Cells were grown on each medium for 36 h, then harvested and resuspended in 20 mM Tris–HCl buffer, pH 8.0. Cells were broken with a 3110BX mini-beadbeater (Biospec Products, Bartlesville, OK, USA) or an Ultrasonic Disruptor UD-201 (Tomy Seiko Co., Ltd., Tokyo, Japan). Cell debris was removed by centrifugation at 12,000×*g* for 10 min at 4°C.

MDH and catechol dioxygenase (XylE) activities were measured according to the methods of Day and Anthony [36] and Springer *et al*. [37], respectively. Protein concentration was determined according to the method of Bradford [38] with a protein assay kit (Bio-Rad Laboratories, Hercules, CA, USA) by using bovine serum albumin as the standard.

### Purification of La^3+^-dependent MDH

Cell-free extracts prepared from cells grown on methanol/Ca^2+^+La^3+^ medium were applied to a PD-10 column (GE Healthcare UK Ltd., Buckinghamshire, UK) pre-equilibrated with 25 mM MES buffer, pH 5.0. The protein was eluted with the same buffer, and applied to a HiTrap Sepharose HP column pre-equilibrated with 25 mM MES buffer, pH 5.5. The protein was eluted with the same buffer containing 1 M NaCl at a flow rate of 1.0 ml/min. The active fractions were pooled and concentrated using an Ultrafree-MC microcentrifuge filter unit with a molecular weight cutoff of 5000 Da (Sigma-Aldrich Co., St. Louis, MO, USA). The purity of the enzyme was confirmed by SDS-PAGE analysis (12.5% polyacrylamide gel). The N-terminal amino acid sequence of the purified protein was determined on a protein sequencer Model 610A (Applied Biosystems, Foster City, CA, USA).

### Determination of La^3+^ and Ca^2+^ contents of the media and the purified enzyme

The contents of La^3+^ and Ca^2+^ in the media were determined using an Agilent 7500cx ICP-MS system (Agilent Technologies, Inc., Santa Clara, CA, USA).

The buffer containing purified enzyme was re-equilibrated in 25 mM Tris-HCl buffer, pH 8.0, using a PD-10 column. The enzyme (2.6 µM) was then incubated with 50 mM EDTA, pH 8.0, at 30°C for 2 h, after which it was desalted and concentrated with an Amicon Ultra-0.5 mL 3 K concentrator (Millipore, Billerica, MA, USA) concentrator and diluted with Milli Q water (Millipore). The La^3+^ and Ca^2+^ contents in the enzyme were determined using an ULTIMA 2 ICP-OES spectrometer (Horiba Ltd., Kyoto, Japan).
